# Arrhythmic Risk Stratification—General Considerations in Specific Scenarios

**DOI:** 10.3390/jcdd11090282

**Published:** 2024-09-07

**Authors:** Marisa Varrenti, Patrizio Mazzone

**Affiliations:** De Gasperis Cardio Center, Niguarda Hospital, 20126 Milan, Italy

Arrhythmic risk stratification is challenging for cardiologists managing patients with different forms of cardiomyopathy, ranging from post-ischemic or non-ischemic cardiomyopathies to systemic diseases with cardiac involvement such as neuromuscular disorders and infiltrative diseases.

International Guidelines try to provide support, especially regarding the prevention of sudden cardiac death, but there are still many gray areas and uncertainties that need to be resolved in this field. 

In this Special Issue, we aimed to collect the experience and the literature data regarding arrhythmic risk stratification in specific clinical scenarios [1,2].

In their work, Noordman et al. (contribution 1) investigated the sex difference in the outcome of patients with implantable cardioverter defibrillators (ICDs) in secondary prevention. Out of 257 patients included in the study, 45 (18%) were women with a mean age of 58 years compared to 65 years in the male population (*p* = 0.003). During the 6.2-year follow-up period, a higher number of appropriate ICD therapies were observed in male than female sex (40% vs. 22%), including appropriate shocks (31% in male vs. 16% in female sex) and appropriate anti-tachypacing (ATP) (33% vs. 18%). No significant differences were observed in regard to inappropriate ICD interventions and the outcome of death. Female sex was negatively associated with the rate of appropriate ICD therapies at both uni- and multivariate analyses. These differences were confirmed in the subgroup of patients with non-ischemic heart disease but not in the subgroup of patients with ischemic heart disease. The authors identify a possible explanation in a hormonal aspect that could be a protective factor in young pre-menopausal women, although this explanation may remain speculative. It must be noted that the observation of a difference in appropriate ICD interventions in the two sexes did not translate into a difference in overall survival.

Concerning arrhythmic risk stratification in patients with ischemic heart disease, Kim et al. reported on data regarding predictors of mortality in patients undergoing coronary artery bypass surgery beyond ejection fraction (contribution 2). The authors investigated heart rate turbulence (HRT) and T-wave alternans (TWA), two non-invasive electrocardiographic markers of cardiac autonomic dysfunction and abnormal ventricular repolarization, respectively, in candidate patients for CABG surgery. Out of 146 patients included in the study, 10% had abnormal HRT and 40% had abnormal TWA. The group with abnormal HRT showed a significant increase in the relative risk of cardiac death and all-cause mortality compared to the group with normal HRT. In contrast, abnormal TWA or left ventricular dysfunction alone showed no association with cardiac death or all-cause mortality. The three variables together, i.e., abnormal HRT and TWA combined with a left ventricular systolic function < 50%, were associated with a 9-fold increase in all-cause mortality and an 87-fold increase in cardiac mortality. The authors concluded that these parameters could be used to screen the high-risk population for adverse outcomes after CABG surgery.

In their literature review, Bonvicini at all analyzed the literature data regarding the risk of arrhythmic events in patients with cardiac amyloidosis, distinguishing among cardiac conduction disorders, supraventricular arrhythmias, and ventricular arrhythmias (contribution 3). About cardiac risk disorders, bradyarrhythmia represents the most common arrhythmic disorder in patients with cardiac amyloidosis, mainly due to the infiltration of amyloid fibrils within the conduction system, which causes fibrosis and atrophy of the sinoatrial and atrioventricular nodes and the development of branch block. At the same time, the affinity of amyloid fibrils for neuronal cells justifies the involvement of the autonomic nervous system, which leads to frequent vasovagal syncope and orthostatic hypotension in these patients. Concerning the risk of rhythm disorders and therefore the need for PM implantation, several studies in the literature have attempted to identify predictors, which have been identified by various authors as the PR interval duration > 200 ms, the QRS interval > 120 ms, and the thickness of the interventricular septum [3,4]. Of particular interest is the finding that each millisecond increase in QRS duration and each millimeter increase in interventricular septal thickness correlates with a 2.6% to 10.6% increase in the risk of PM implantation. Atrial fibrillation represents the most frequent tachyarrhythmia in patients with cardiac amyloidosis and the relevance of its early diagnosis is mainly related to the prevention of related ischemic events.

There are several mechanisms underlying the development of ventricular arrhythmic events in patients with cardiac amyloidosis, including the presence of amyloid fiber substances and the areas of interstitial fibrosis that create the substrate for the development of possible arrhythmic circuits.

However, the indication for defibrillator implantation in primary prevention in this patient group is still a major debate in the literature. 

According to the latest ESC guidelines on the prevention of sudden cardiac death, ICD implantation should be considered in patients with light-chain amyloidosis or transthyretin cardiac amyloidosis who have hemodynamically intolerable VT (Class IIa recommendation, Level of Evidence C) [1]. 

While the latest ESC guideline on cardiomyopathies emphasizes that the role of ICD in primary prevention in this group of patients is not clearly known [2], in the work by Bonvicini et al., the authors propose an operative flow chart that considers life expectancy, left ventricular function, and the pattern of ventricular arrhythmias and syncope in making decisions on whether to implant an ICD in primary prevention in patients with cardiac amyloidosis.

Another area of interest regards patients affected by neuromuscular diseases. In more detail, Russo et al. propose an operative flow chart regarding the management of arrhythmic risk in patients with myotonic dystrophy type 1 (contribution 4).

Myotonic dystrophy type 1 is a hereditary disorder with cardiac involvement in 80% of cases characterized by fibroadipose replacement of the myocardium and cardiac conduction system. This then leads to the development of conduction system disorders, atrial fibrillation, and ventricular arrhythmias, with the need therefore to define a precise strategy in the prevention of sudden cardiac death.

Data from the literature have identified the following as independent predictors of sudden cardiac death: the presence of cardiac rhythm disorders, PR interval ≥ 240 ms, QRS duration ≥ 120 ms, second- or third-degree atrioventricular block, left bundle-branch block, age, and family history of sudden cardiac death [5,6,7].

In their work, Russo et al. propose a multidisciplinary and multi-parametric approach, involving collaboration between cardiologists and neurologists and the use of tools including clinical history, electrocardiogram, echocardiogram, cardiac MRI, and electrophysiological study to intercept patients at high risk of rhythm disturbances and therefore sudden cardiac death at an early stage.

Finally, in their work, De Gregorio et al. (contribution 5) investigated the incidence of subclinical atrial fibrillation (SAF) using prolonged continuous electrocardiographic monitoring (median 13.5 days of monitoring) in a group of 119 patients. SAF episodes were found in 19 patients (16%) of which 7.6% were longer than 5 min, underscoring how prolonged electrocardiographic monitoring, e.g., using small indomitable devices, can help to diagnose the presence of AF in patients at risk but without a previous history of arrhythmias.
